# Contribution of Anatomy in Forensic Age Evaluation: A Systematic Review

**DOI:** 10.7759/cureus.55080

**Published:** 2024-02-27

**Authors:** Rohin Garg, Sanjay Gupta, Simmi Mehra, Utsav Parekh

**Affiliations:** 1 Anatomy, All India Institute of Medical Sciences, Rajkot, Rajkot, IND; 2 Forensic Medicine and Toxicology, All India Institute of Medical Sciences, Rajkot, Rajkot, IND

**Keywords:** sex, anthropology, age, forensic, bone age, fingerprints, forensic age estimation, teeth, human anatomy, acetabulum

## Abstract

The field of forensic anthropology is characterised by its ongoing development and growth. Forensic anatomy is a burgeoning discipline that focuses on the analysis and identification of both preserved and unpreserved human body parts, both in deceased individuals and the living. This subject plays a crucial role in establishing the four key factors of forensic anthropology, namely sex, age, race, and height. The objective of this research endeavour was to evaluate the significance of anatomical information in the process of forensic age estimation. The researchers established the inclusion criteria in accordance with the globally recognised Population, Intervention, Comparison, Outcome* *(PICOS) framework, as advised by the Preferred Reporting Items for Systematic Reviews and Meta-Analyses (PRISMA) recommendations. The research included many methodologies in order to ascertain the age. Upon conducting a comprehensive review of the existing literature pertaining to anatomical knowledge in the field of forensic age estimate, we have identified many notable applications. These include the utilisation of various anatomical features such as the dental pulp chamber, fingerprints, acetabulum, sternal end of the fourth rib, as well as hand and wrist bones for the purpose of age estimation. It is important for anatomists and other forensic scientists to engage in collaborative efforts to facilitate the exchange of ideas and ensure thorough investigations. This cooperation is particularly crucial in areas where anatomical sciences play a significant role in forensic science and investigation. Nevertheless, in order to mitigate the potential for estimating error, it is still advisable to use a multi-factorial evaluation approach that involves examining many body areas.

## Introduction and background

The field of forensic anthropology is characterised by its ongoing evolution and expansion. Forensic anatomy is a burgeoning discipline that aims to ascertain, analyse, and distinguish preserved or unconserved anatomical elements of human remains as well as live individuals. This process is crucial in establishing the fundamental components of forensic anthropology, namely sex, age, race, and height. Furthermore, it investigates the underlying factors leading to mortality [1].

The responsibilities of a forensic anthropologist encompass the conventional examination of human skeletal remains with the objective of establishing identification, such as constructing a biological profile, analysing trauma, and reconstructing facial features. However, the scope of their work has expanded to encompass the identification of living individuals, such as determining whether a person has attained the age at which they can be held criminally responsible. The growth of the field, in conjunction with advancements in technology, has resulted in a tangible rise in the creation of new techniques and the enhancement of pre-existing ones [2].

The age of living people must be estimated in a variety of forensic situations pertaining to legal issues, including child pornography, migration, classifying suspects as juveniles or adults, and age progression in cases of missing persons. Numerous challenges arise from the varying country-specific interpretations of legal adulthood with respect to persons whose age is undocumented [3].

The historical viewpoint pertains to the assessment of the shared history of human anatomy and forensic anthropology. The intrinsic connection between forensic anthropology and human anatomy is readily apparent in the analytical methodologies and the progression of every forensic anthropological investigation. Indeed, the undeniable connection between these two scientific disciplines is evident. The understanding of human anatomy is fundamental in the field of forensic anthropology [4]. The objective of this research endeavour was to evaluate the significance of anatomical information in the process of forensic age estimation.

## Review

Materials and methods

Data Sources

This study encompasses research undertaken globally on the significance of anatomical information in forensic age assessment, specifically focusing on studies published from 2000 to June 2023. The primary focus of consideration revolved on the assessment of age based on anatomical sites. The review only included papers that were published in peer-reviewed publications in English language. A comprehensive review of the literature was conducted using several databases, including PubMed, Scopus, Embase, Google Scholar, clinicaltrials.gov, and the Cochrane Library. The search included publications from the year 2000 to March 2023. The search phrases used pertained to the field of forensic age estimate.

Study Selection

The study included an assessment of the significance of anatomical information in forensic age estimation, as shown in several research investigations. The reported role of various human anatomical regions in forensic age estimation was assessed. The analysis included investigations of all the aforementioned therapies, including those that did not have a comparator group. The data pertaining to the determination of gender based on anatomical differences was omitted.

The evaluation included a comprehensive range of experimental research, observational studies, and case series that documented the methodologies and results pertaining to the aforementioned techniques. Any articles not fulfilling inclusion criteria or any articles that presents repetitive data from other included articles were excluded.

The references of chosen articles were examined in order to identify any relevant sources that were not captured by the search databases. Additional references were not acquired using a free internet search using Google due to the huge quantity of research available.

Data extraction and analysis

The data contained in the studies were carefully reviewed and manually extracted into a standardised data extraction form.

The search approach used in this study consisted of three distinct stages: first assessment of titles, subsequent evaluation of abstracts, and final selection of articles for comprehensive examination of their complete texts. Two reviewers independently classified each of the articles that were selected from the database search; disagreements arose during the selection process and were discussed until a mutually acceptable conclusion was reached. Based on the reviewers' consensus, articles that did not meet the predetermined inclusion criteria were rejected. The reviewers evaluated the abstracts of the papers they had picked in the second round separately, and the complete articles of those that made it to the final analysis were retrieved.

The title and abstract of the citations were appended to a designated endnote library. After that, duplicate entries were removed to create a final compilation of papers to be evaluated for inclusion in the study. Efforts were undertaken to acquire complete-text articles for all the studies that were selected, and a comprehensive evaluation was conducted to ensure compliance with the predetermined criteria for inclusion and exclusion. During the third and final step, a comprehensive analysis was conducted on the whole text of the acquired articles.

The quality rating criteria created by Fowkes and Fulton were used to assess the methodological rigour of the studies included in the systematic review [5].

Results

Upon conducting a comprehensive review of relevant scholarly material pertaining to the assessment of anatomical knowledge for forensic age estimate, we have identified many notable applications. These include the use of dental pulp chamber analysis, fingerprint examination, acetabulum assessment, evaluation of the sternal end of the fourth rib, as well as the examination of hand and wrist bones for the purpose of age estimation.

Applications of Dental Pulp Chamber in Age Estimation

Upon conducting a comprehensive review of the existing literature pertaining to anatomical information in the context of forensic age estimate, we have identified many notable applications. These include the utilisation of dental pulp chamber, fingerprints, acetabulum, sternal end of the fourth rib, as well as hand and wrist bones for the purpose of estimating an individual's age. In this study, Abdinian et al. [6] examine the connection between the pulp-to-tooth volume ratio in the front teeth and the patient's age. They offer formulas for calculating age based on pulp-tooth volume ratio using cone beam computed tomographic (CBCT) data. The participants ranged in age from 16 to 69, with a mean age of 40.6 ± 12.74 years. The ratio of pulp volume to tooth volume in the maxillary teeth was often found to be greater compared to the mandibular teeth. A notable negative correlation between age and the ratio of pulp volume to tooth volume was seen for all anterior teeth. The study found that there was a significant positive connection between age and the ratio of pulp-tooth volume for the mandibular central incisor, indicating that as age increased, the pulp-tooth volume ratio also increased. Conversely, the correlation between age and the pulp-tooth volume ratio for the mandibular lateral incisor was found to be weak, suggesting that age had less influence on this particular tooth. The accuracy of age assessment was shown to be highest in females when using the maxillary central incisors (p < 0.001), whereas in males, the most accurate estimates were obtained by considering the mandibular central incisors and maxillary canines (p = 0.003) [7].

Applications of Fingerprints in Age Estimation

A fingerprint is a distinctive imprint created by the friction ridges, which exhibit almost parallel patterns with a consistent crest-to-crest wavelength. The observed pattern is primarily characterised by core characteristics, including whorls, loops, arches, and triradii. Upon further examination, several other flaws become apparent, including ridge ends, ridge bifurcations, and island ridges, among others. The classification and spatial arrangement of these dislocations serve as the fundamental factors contributing to the distinctiveness of fingerprints. The distinctiveness and permanence of fingerprints throughout the course of an individual's lifetime are among the characteristics used for personal identification [8].

The characteristics of epidermal ridges and their organisation, known as dermatoglyphic patterns, possess many attributes that are indicative of an individual's biological traits. There are statistically significant variations in dermatoglyphic characteristics seen across different sexes, ethnic groupings, and age categories. The determination of dermatoglyphics and their components is influenced by both environmental and hereditary factors, but the configuration of ridges stays consistent throughout the course of an individual's lifespan. In a theoretical framework, it is conceivable to use human fingerprints found on archaeological artefacts in a manner analogous to skeletal remains, with the aim of estimating several interrelated biological characteristics of the individuals responsible for leaving the fingerprints [9].

In research undertaken by Gutiérrez-Redomero et al. [10], an examination was carried out on a sample of Amerindians from the Mataco-Mataguayo people. The findings indicated a negative correlation between age and ridge density, with greater ridge density seen on the distal (radial and ulnar) sections compared to the proximal sides across all age groups.

Age Estimation by Acetabulum

The acetabular area is often seen and sufficiently maintained in skeletal remains of mature humans [11]. The research conducted by Calce et al. [12] aimed to assess the accuracy and precision of a skeletal age assessment technique that used the acetabulum. The findings of the investigation revealed an observed error of eight years in this particular approach. The observed bias direction suggests that the acetabulum approach has a tendency to underestimate age. The age groups of 46-65 and 76-90 years demonstrate the least amount of error, with an inaccuracy of 0.2. This finding implies that the proposed approach might be suitable for adults aged 40 years and above. The results indicate that 83% of the age estimates had a margin of error of ±12 years when compared to the known age. Similarly, 79% of the age estimates had a margin of error of ±10 years, while 62% had a margin of error of ±5 years when compared to the known age. The primary constraint of this method for forensic applications is in the identification of an appropriate reference population. However, another work conducted by Belghith et al. [13] used postmortem CT images of the acetabulum and employed global illumination rendering (GIR) with computed tomography to determine age. The inter-observer and intra-observer reproducibility for the three variables, as measured by intraclass correlation (ICC), showed a strong level of agreement (ICC ranging from 75.6% to 90.8% and 89.3% to 95.8%, respectively). Similarly, the total score exhibited a high level of agreement (ICC of 93.5% and 95%, respectively). There was a strong correlation seen between the three factors and the overall score, with respect to different age groups. The overall score demonstrated a predictive accuracy over 85% for those aged below 40 and beyond 70 years. In a study conducted by Rissech et al. (2006), [11] the age at death of adult males in the Portuguese population was estimated using the acetabulum. The researchers employed Bayesian inference to predict the age of identified specimens and found a strong correlation between the acetabular criteria and age. Additionally, the study reported a high accuracy rate of 89% in the Bayesian prediction of age. The estimated age of the specimens was found to have consistent accuracy across all age groups.

Age Estimation from the Sternal End of the Fourth Rib

The fourth rib's sternal end has been recognised as a valuable anatomical location for determining the sex and age-at-death of skeletal remains [14]. The determination of age in deceased individuals by the examination of post-mortem "chest plate" using conventional radiography is a method that includes the radiographic evaluation of ossification patterns around the sternum and rib ends [15].

In their study, Muñoz et al. [14] found that the average percentage of accurate age assignments for the fourth rib test within its original age ranges was relatively low (31.21-43.17%). Furthermore, their research revealed a tendency towards underestimating and moderate to high levels of error in age determination.

However, Monum et al. [15] showed a greater level of accuracy. This research investigated the use of three-dimensional post-mortem CT scans of the chest plate in estimating the age at the time of death. A total of five scores were assessed to evaluate the ossification of chest plates. The ossification of the second to seventh costal cartilages at the rib and sternal ends, as well as the ossification of the first costal cartilage (also known as OF), were included in these scores (referred to as OR and OS, respectively), the fusion of the manubriosternal joint and the fusion of the xiphisternal joint. The study revealed that the variable "OS" had the strongest link with age, but the variable "FM" did not demonstrate a statistically significant correlation. The accuracy rate within the first standard error of estimate was found to be 57.69% for males and 70.83% for women.

Bone Age by Visualization of Hand and Wrist Bones

A key component of forensic age estimation is the utilization of X-ray examination for hand bone age evaluation. The user did not provide any text to rewrite. The evaluation of bone age (BA) may provide significant clinical insights into estimating skeletal maturity, particularly in the context of diagnosing endocrinological issues and growth abnormalities. The disparity seen between bone age and chronological age serves as an indicator of irregularities in skeletal growth. Radiologists in clinical settings engage in the practise of bone age assessment (BAA) by means of analysing X-ray images of the left hand and wrist. In the field of clinical practise, two BAA approaches, namely the Greulich and Pyle (GP) method and the Tanner Whitehouse method (TW2), have been extensively used throughout history [16,17].

In their study, Widek et al. [16] used the GP atlas to analyse magnetic resonance (MR) scans of the hand and wrist. Their objective was to provide a set of reference values that could be utilised for evaluating the age of hand bones. The acquisition of 3T hand MR images was performed utilising 3D gradient echo sequences, VIBE and DESS techniques. The comparison of the readers' chronological ages revealed a discrepancy of 0.37 and 0.54 years, respectively. The cross-validated transition analysis yielded an average inaccuracy of -0.28 years in estimating age. The research concluded that using MR images of hand bones with the GP atlas as an alternative to ionising radiation allows for routine and reliable age calculation.

In their study, Pan et al. [17] developed a comprehensive preprocessing pipeline that uses deep learning techniques to automate the detection and segmentation of the hand and wrist. This pipeline also includes image standardisation and utilises pretrained deep CNNs and a high-efficiency regression model for BAA.

Soliman et al. [18] estimated age of epiphyseal union around wrist joint and its correlation with chronological age and reported that the distal epiphyses of radius and ulna fused completely at 18-19 years in females and 19-20 years in males. With every stage of union, the mean age grew and changed gradually, with a statistically significant difference between the equivalent averages of males and females. Epiphyseal union occurred one year earlier in females than in males. Male accuracy on the ROC curve was 90.9%, while female accuracy was 90.6%. Elamin et al. [19] carried out research in Khartoum, Sudan, to characterise the bone-specific maturity for growing hand and wrist bones in people. The presentation includes maturity data for both male and female participants for the phalanges, metacarpals, carpals, radius, and ulna. For all stages, the median age of females was lower than that of males.

Age Estimation From the Pelvic Bone

Bartolini et al. [20] evaluated the applicability of three different age estimation methods on pelvic radiographs of Italian individuals between 10 and 25 years of age. Since the staging method is unaffected by ossification variances, it is easier to apply than the Risser method. It was influenced by the Kreitner and Kellinghaus methods (KK-MS). Every technique demonstrated excellent repeatability and reproducibility. The Cameriere-inspired area measurement method has a 12-to-20-year applicability range, although the statistical analysis revealed only a moderate connection with age. Several mathematical techniques were used by Kotěrová et al. [21] to estimate age more accurately and consistently. The hip bone's auricular surface and pubic symphysis age-related alterations were assessed using a multiethnic dataset (n=941). Nine distinct mathematical techniques were investigated by two research teams. Multi-linear regression produced the greatest results, followed by the Collapsed regression model, with RMSE values of 12.1 and 12.2 and MAE values of 9.7 and 9.9 years, respectively. Decision tree models' average accuracy varied from 30.7% to 72.3%; the model that solely used the PUSx indicator performed the best. Martins et al. [22] estimated the age at death using four distinct osteological series from Portugal, Great Britain, South Africa, or the United States (European provenance) and two indicators (pubic symphysis and the sacro-pelvic surface of the ilium). Using Jackknife methods and kernel density procedures, the different probability distributions are estimated from training data. Next, the posterior distribution-from which point and interval estimates may be derived-is generated using Bayes' theorem. By using this statistical technique, the estimates' bias is reduced to less than 70% of what the original method produced. If the person's sex is known, this reduction increases to 52%, and it results in an age for each person that enhances the assessment of their age at death.

Age Estimation From the Femur

Our femur variables- diapyseal length, diaphyseal length plus distal epiphysis, maximum length, and vertical diameter of the head- were studied by Rissech et al. [23] in order to assess their significance and ability to determine age and sex over the course of a person's entire life. The data was analyzed using polynomial regression. For all four metric variables, there were significant correlations found between age and femoral size. In a Thai population, aspartic amino acid racemization from a femur was used by Monum et al. [24] to assess age prediction techniques. Using high-performance liquid chromatography (HPLC), 40 femur bones from 24 males and 16 females were subjected to a Dextro/Levo (D/L) ratio study. The results showed a 0.8316 connection between the D/L ratio and age. The correlation coefficients in the male and female samples were 0.912 and 0.716, respectively, higher in the male sample. For every sample, the standard error of estimation was 11.01 years. In a work by Pham et al. [25], a fully automated method for predicting age was presented by evaluating 3D scans of the femur and mandible using deep learning. 814 post-mortem computed tomography scans, spanning the ages of 20 to 70, were gathered from the National Forensic Service in South Korea, comprising 619 males and 195 women. For every scan, a number of preprocessing stages were used to normalize the picture and adjust the intensity in order to produce 3D voxels that faithfully depict these components. The 10-fold cross-validation approach was used to assess the suggested method's accuracy. The first cross-validation results indicated a mean absolute error of 5.15 years and a concordance correlation coefficient of 0.80, indicating the promise of the proposed strategy. Future age assessments could make use of the suggested method, which is probably quicker and possibly more accurate.

Abdulai et al. [26] used osteometric data from the proximal femur to estimate the age and sex of an adult population in Ghana. Although the confidence interval (95%CI) was larger than anticipated, there was no discernible difference between the estimated age of males using hip axis length-left (HALL) and their chronological age.

Recent research findings have indicated that knee magnetic resonance imaging (MRI) may also be able to determine the majority beyond a reasonable doubt [27]. By creating and utilizing a novel stage classification, Vieth et al. [28] investigated the feasibility of establishing majority by a morphology-based assessment of the epiphyseal-diaphyseal fusion by 3.0 T magnetic resonance imaging (MRI). A 3.0 T MRI scanner was used to obtain a T1-weighted (T1-w) turbo spin-echo sequence (TSE) and a T2-weighted (T2-w) TSE sequence with fat suppression by spectral pre-saturation with inversion recovery (SPIR). After sorting through the data, a five-stage categorization scheme was developed as a hypothesis. This categorization was then applied to evaluate the photos. The pertinent statistics were identified, the sex differences were examined, and the intra- and interobserver agreements were ascertained. By using the provided MRI categorization, a 3.0 T knee joint MRI can be used to assess whether a person has reached adulthood in either sex. As the study revealed minimum ages above the age of 18 years for this stage (20.40 years in males and 20.60 years in females), Chitavishvili et al. [27] confirmed that MRI of the distal femoral epiphysis (DFE) is suitable to determine majority in both sexes when stage 6 is present (Ossification stages of the DFE were determined by means of the classification system by Vieth et al. [28]). Therefore, it looks like a practical substitute for forensic age estimation technique in the near future will be the examination of the knee using routine MRI.

Age Estimation From the Humerus

According to Singh et al. [29], female epiphyses at the lower end of the humerus fuse at the age of 16. The final epiphysis to fuse at the lower end of the humerus in both males and females is the medial epicondyle. With the exception of the medial epicondyle around age 16, all of the lower humerus's epiphyses are united in males. Though the range varied, which can be attributable to a variety of causes, including but not limited to geographic variation and nutritional considerations, the sequence of fusion at the lower end of the humerus was nearly identical to that of other workers.

The fusion of the epiphyseal center of the lateral epicondyle with the capitulum, the fusion of the epiphyseal center of the capitulum with the trochlea, the fusion of the conjoint epiphysis with the shaft, and the fusion of the epiphyseal center of the medial epicondyle with the shaft were all observed between 12 and 15 years, according to a radiological study of the lower end of the humerus by Choudhary et al. [30].

Age Estimation From the Elbow

In their analysis of dorso-volar projected normal radiographs of the elbow and wrist joints, Ominde et al. [31] discovered that the head of the radius appeared at six to seven and five to six years in males and females, respectively, and the epiphyseal plate of the distal radius was seen in age groups three to four and two to three years, the distal ulna in age groups nine to 10 and eight to nine years, and the medial epicondyle at eight to nine and seven to eight years. The head of the radius fused at 16-17 years, the distal radius at 18-19 and 17-18 years, the distal ulna at 19-20 and 18-19 years, and the medial epicondyle at 17-18 and 16-17 years, for both boys and girls.

In her research, Jacquiline [32] examined the ages at which the secondary ossification centers surrounding the elbow and wrist joints fused. She discovered that, in males, the fusion of each center began between the ages of 15 and 16 and was finished by the age of 19; in females, it began between the ages of 15 and 16 and was completed around the age of 16 to 17. In the wrist joint, fusion begins in males at the age of 16-17, while in females, it completes at the age of 18-19, fusing both the ulna and the lower end of the radius. The fusion of ossification centers research indicates that the fusion of ossification centers around the elbow and wrist joints occurs one to two years earlier in females than in males.

The Sauvegrain method uses four anatomical landmarks of the elbow: the lateral condyle, trochlea, olecranon apophysis, and proximal radial epiphysis. It is based on a 27-point scoring system. The scores for these structures are summed, and a total score is determined. A graph is then used to determine the skeletal age [33]. Diméglio et al. [33] revealed that the skeletal age determination from radiographs of the elbow was more precise because a clear semiannual age determination was possible. The correlation between the methods of Sauvegrain et al. and Greulich and Pyle was good and it was reported that the modified method of Sauvegrain et al. is simple, reliable, and reproducible, and it complements the Greulich and Pyle atlas.

Assessment of Age From Epiphyseal Fusion at the Wrist and Ankle

The distal ulna and radius of males and females started to appear at the age of nine to 16, while the distal tibia and fibula of males started to appear at the age of nine to 17, according to Ebeye et al.'s assessment of anterior-posterior and lateral radiographs of the distal end of bones at the wrist and ankle [34]. From the ages of 15 to 19, girls showed complete fusion, suggesting that they had consistently developed epiphyseal fusion earlier than boys. At the age of 19, boys achieved complete epiphyseal fusion at the distal end of the fibula and tibia.

Gajera et al. [35] reported that fusion of epiphysis of distal end of radius occurs at 21 years in males and at 20 years in females. The fusion of epiphysis of distal end of ulna occurs at 21 years in males and 19 years in females. Thus in females, the ossification centers of distal end of radius and ulna occurs earlier than in males by one to three years. The distal radius and distal ulna's epiphyseal development was evaluated using a five-stage ossification classification system by Baumann et al. [36] as follows: 1: epiphysis not ossified; 2: epiphysis ossified, nonunion of the epiphysis and metaphysis; 3: partial union of the epiphysis and metaphysis; 4: complete union of the epiphysis and metaphysis, visible epiphyseal scar; 5: complete union of the epiphysis and metaphysis, nondiscernable epiphyseal scar). It was established that male subjects with an ossification stage 4 of the radius or ulna and female subjects with an ossification stage 5 of the radius were at least 14 years old. The occurrence of ossification stage 5 of the radius proves that a male individual has reached the age of 18 years.

Assessment of Age From Cranial Sutures

Hershkovitz et al. [37] studied the extent of the sagittal suture closure in 3,636 skulls from the Hamann-Todd and Terry collections. Sagittal suture closure was found to be age-independent and sexually biased. The wide confidence intervals (for age) appear to preclude meaningful application of suture status for age determination. No correlation was found with the tested biologic stressors.

Role of Ultrasonography in Age Estimation

Bilgili et al. [38] determined the ultrasonographic version of the Greulich-Pyle atlas in assessing skeletal age. The bone ages estimation from plain radiography and hand and wrist ultrasonography charts interpreted by use of the Greulich-Pyle atlas were significantly correlated. It was found that this method is a valid alternative and is highly correlated to plain radiography for bone age estimation. This enables estimation of skeletal age in ultrasonography departments easily without exposing the patient to radiation.

The role of anatomical knowledge in age estimation is summarised in Table 1.

**Table 1:** Contribution of Anatomy in Forensic Age Evaluation

Author (year)	Country	Anatomical location	Age estimation
Soliman KE et al. (2023) [18]	Saudi Arabia	Wrist joint	With every stage of union, the mean age grew and changed gradually, with a statistically significant difference between the comparable means for males and females.
Elamin F et al. (2023) [19]	Sudan	Hand-wrist	The presentation includes maturity data for both male and female participants for the phalanges, metacarpals, carpals, radius, and ulna. In every stage, the median age of girls was lower than that of males.
Abdinian M et al. (2021)[7]	Iran	Pulp Chamber	An accurate way to estimate age may be to measure the pulp volume to tooth volume ratio in the mandibular central incisors, maxillary lateral incisors, and maxillary canines of males, as well as in the maxillary and mandibular central incisors, maxillary lateral incisors, and maxillary and mandibular canines of females.
Ebeye OA et al. (2021) [33]	Nigeria	Wrist and ankle	Females demonstrated complete fusion from 15 to 19 years of age, demonstrating that females have consistently developed epiphyseal fusion at an earlier age relative to males. Males attained complete epiphyseal fusion at the distal end of the tibia and fibula at age 19.
Belghith M et al. (2021) [13]		Acetabulum	The prediction accuracy exceeds 85% for those below the age of 40 and those over the age of 70.
Monum T et al. (2020) [14]	Japan	Chest plate	The study revealed that there was a strong positive connection between age and sternal ends, indicating that as age increased, the measurement of sternal ends also increased. However, no significant association was seen between age and the manubriosternal joint.
Baker A et al. (2019) [6]	India	Pulp Chamber	The number of odontoblasts exhibits a decline with advancing age, with a statistically significant difference seen between each successive decade of life. The thickness of collagen fibres exhibited a positive correlation with increasing age, with an accuracy rate of 85.7%.There is a notable decline in both mean blood vessel area and microvessel density (MVD) as individuals age.
Ominde BS et al. (2019) [31]	Nigeria	Radius	The distal radius's epiphyseal plate appeared in age groups 3–4 and 2-3 years, the distal ulna in age groups 9–10 and 8–9 years, the medial epicondyle in age groups 8–9 and 7-8 years, and the head of the radius in age groups 6–7 and 5–6 years, respectively, in both males and females.
Monum T et al. (2019) [24]	Thailand	Aspartic amino acid racemization from a femur	The correlation coefficients in the male and female samples were 0.912 and 0.716, respectively, higher in the male sample. For every sample, the standard error of estimation was 11.01 years.
Muñoz A et al. (2018) [14]	Mexico	The sternal end of the fourth rib	The examination of the initial age ranges for the fourth rib reveals a rather low average rate of accurate age determinations (ranging from 31.21% to 43.17% for females and males, respectively), indicating a tendency towards underestimating and a moderate to high level of inaccuracy.
Bartolini V et al. (2018) [20]	Italy	Iliac Crest	Every technique demonstrated excellent repeatability and reproducibility. Since the staging method is unaffected by ossification variances, it is easier to apply than the Risser method. It was influenced by the Kreitner and Kellinghaus methods (KK-MS).
Martins R et al. (2012) [22]	Portugal, Great-Britain, South Africa or USA (European origin).	Pubic symphysis and the sacro-pelvic surface of the ilium	By using a statistical approach, the bias of the estimations is reduced to less than 70% of what the original method produced. If the person's sex is known, this reduction increases to 52%, and it results in an age for each person that enhances the assessment of their age at death.
Calce SE et al. (2011) [12]	Canada	Acetabulum	The age groups of 46–65 and 76–90 years demonstrate the lowest level of inaccuracy, with a margin of error of 0.2. This approach is suitable for those who are 40 years old and above. A total of 83% of the age estimates exhibited a margin of error within ±12 years of the known age. Similarly, 79% of the age estimates displayed a margin of error within ±10 years of the known age. Furthermore, 62% of the age estimates shown a margin of error within ±5 years of the known age.
Gutiérrez-Redomero E et al. (2011) [10]	Spain,Argentina	Fingerprints [Ridge density (RD), the number of digital ridges per unit are]	At all ages, RD was highest in the distal (radial and ulnar) regions and reduced with age, with the proximal sides showing the greatest decrease in RD.
Kralik M et al. (2003) [9]	Czech Republic	Fingerprints	When a person's mean epidermal ridge breadth (MRB) measurement is less than 0.39 mm, they are considered sub-adults younger than fifteen. In contrast, adult males are the only ones with MRB values greater than 0.52 mm.

## Conclusions

The expeditious and non-intrusive technique for age estimation warrants examination in comparison to traditional approaches, as it has potential for accurately determining the age at the time of death. Nevertheless, in order to mitigate the potential for estimating error, it is still advisable to use a multi-factorial evaluation approach that incorporates examination of many body parts. In conclusion, it can be said that the inclusion of anatomical sciences is of utmost importance in the fields of forensic science, teaching, and investigations. Consequently, it is imperative to provide anatomists with a keen interest in forensics with the opportunity to actively engage in these domains. This will eventually foster opportunities and facilitate cooperation between anatomists and other forensic scientists, enabling the exchange of ideas and promoting appropriate investigations. It emphasizes the significance of anatomical sciences in the field of forensic research and investigation.
